# Nonstructural protein Pns4 of rice dwarf virus is essential for viral infection in its insect vector

**DOI:** 10.1186/s12985-015-0438-6

**Published:** 2015-12-09

**Authors:** Qian Chen, Linghua Zhang, Hongyan Chen, Lianhui Xie, Taiyun Wei

**Affiliations:** Fujian Province Key Laboratory of Plant Virology, Institute of Plant Virology, Fujian Agriculture and Forestry University, Fuzhou, Fujian 350002 PR China

**Keywords:** Rice dwarf virus, Leafhopper vector, Pns4 minitubules, Viroplasm, RNA interference

## Abstract

**Background:**

Rice dwarf virus (RDV), a plant reovirus, is mainly transmitted by the green rice leafhopper, *Nephotettix cincticeps*, in a persistent-propagative manner. Plant reoviruses are thought to replicate and assemble within cytoplasmic structures called viroplasms. Nonstructural protein Pns4 of RDV, a phosphoprotein, is localized around the viroplasm matrix and forms minitubules in insect vector cells. However, the functional role of Pns4 minitubules during viral infection in insect vector is still unknown yet.

**Methods:**

RNA interference (RNAi) system targeting Pns4 gene of RDV was conducted. Double-stranded RNA (dsRNA) specific for Pns4 gene was synthesized *in vitro*, and introduced into cultured leafhopper cells by transfection or into insect body by microinjection. The effects of the knockdown of Pns4 expression due to RNAi induced by synthesized dsRNA from Pns4 gene on viral replication and spread in cultured cells and insect vector were analyzed using immunofluorescence, western blotting or RT-PCR assays.

**Results:**

In cultured leafhopper cells, the knockdown of Pns4 expression due to RNAi induced by synthesized dsRNA from Pns4 gene strongly inhibited the formation of minitubules, preventing the accumulation of viroplasms and efficient viral infection in insect vector cells. RNAi induced by microinjection of dsRNA from Pns4 gene significantly reduced the viruliferous rate of *N. cincticeps*. Furthermore, it also strongly inhibited the formation of minitubules and viroplasms, preventing efficient viral spread from the initially infected site in the filter chamber of intact insect vector.

**Conclusions:**

Pns4 of RDV is essential for viral infection and replication in insect vector. It may directly participate in the functional role of viroplasm for viral replication and assembly of progeny virions during viral infection in leafhopper vector.

## Background

Viruses in the family *Reoviridae* are thought to replicate and assemble within cytoplasmic, nonmembranous structures called viroplasms [1]. For animal reoviruses, including reoviruses, rotaviruses and bluetongue virus (BTV), viral dsRNA, mRNA and proteins, and host components such as ribosomes, protein-synthesis machinery and chaperones aggregate within the viroplasm matrix [1–4]. Furthermore, some host components such as ribosomes, membrane components, mitochondria and microtubules have been observed to accumulate at the periphery of the viroplasm matrix [1, 3, 4]. The host components at the periphery of the viroplasm may be utilized to facilitate viral replication and assembly of progeny virions during viral infection in host cells.

Rice dwarf virus (RDV), a phytoreovirus in the family *Reoviridae*, is mainly transmitted by the leafhopper vector, *Nephotettix cincticeps*, in a persistent-propagative manner [5]. RDV is an icosahedral and double-layered spherical virion of approximately 70 nm in diameter. The RDV genome consists of 12 double-stranded RNA (dsRNA) segments (S1-S12), encoding at least seven structural proteins (P1, P2, P3, P5, P7, P8 and P9) and five nonstructural proteins (Pns4, Pns6, Pns10, Pns11 and Pns12) [6, 7]. The viral core particle is composed of the inner core protein P3, which encloses P1, a putative RNA polymerase, P5, a guanylyltransferase, and P7, a protien with RNA-binding activity [8–11]. The outer capsid shell is composed of the major outer capsid protein P8, and the minor outer capsid proteins P2 and P9 [12–16]. The functions of nonstructural proteins in the insect vector have been determined using continuous cell cultures derived from *N. cincticeps*. Pns10 assembles into tubules that package virions to facilitate viral intercellular spread [17–21]. Pns6, Pns11 and Pns12 aggregate together to form the viroplasm matrix for viral replication and progeny virions assembly [22]. Our recent report shows that Pns12 of RDV is a principal regulator for viral replication and infection in its insect vector [23]. Pns4, a phosphoprotein, is localized around the viroplasm in continuous cell cultures of *N. cincticeps* [24–26]. The combination of immunofluorescence and immunoelectron microscopy has revealed that at the early stage of viral infection, Pns4 accumulates at the periphery of the viroplasm, and at later stages, it associates with novel minitubules of approximately 10 nm in diameter [26]. In the viruliferous leafhopper, Pns4 assembles into bundles of minitubules that surround the viroplasm matrix [26]. However, whether the formation of the Pns4 minitubule is essential for viral replication and infection in insect vector cells is undetermined yet.

In this study, the functional role of Pns4 in the viral infection cycle was investigated using the system of RNA interference (RNAi) in cultured leafhopper cells *in vitro* and insect body *in vivo*. Our results showed that Pns4 of RDV is crucial for viral infection and replication in insect vector, which may play a role in viral replication and assembly of progeny virions in leafhopper vector.

## Results

### RNAi induced by dsPns4 inhibits the replication and infection of RDV in cultured insect vector cells

**Fig. 1:**
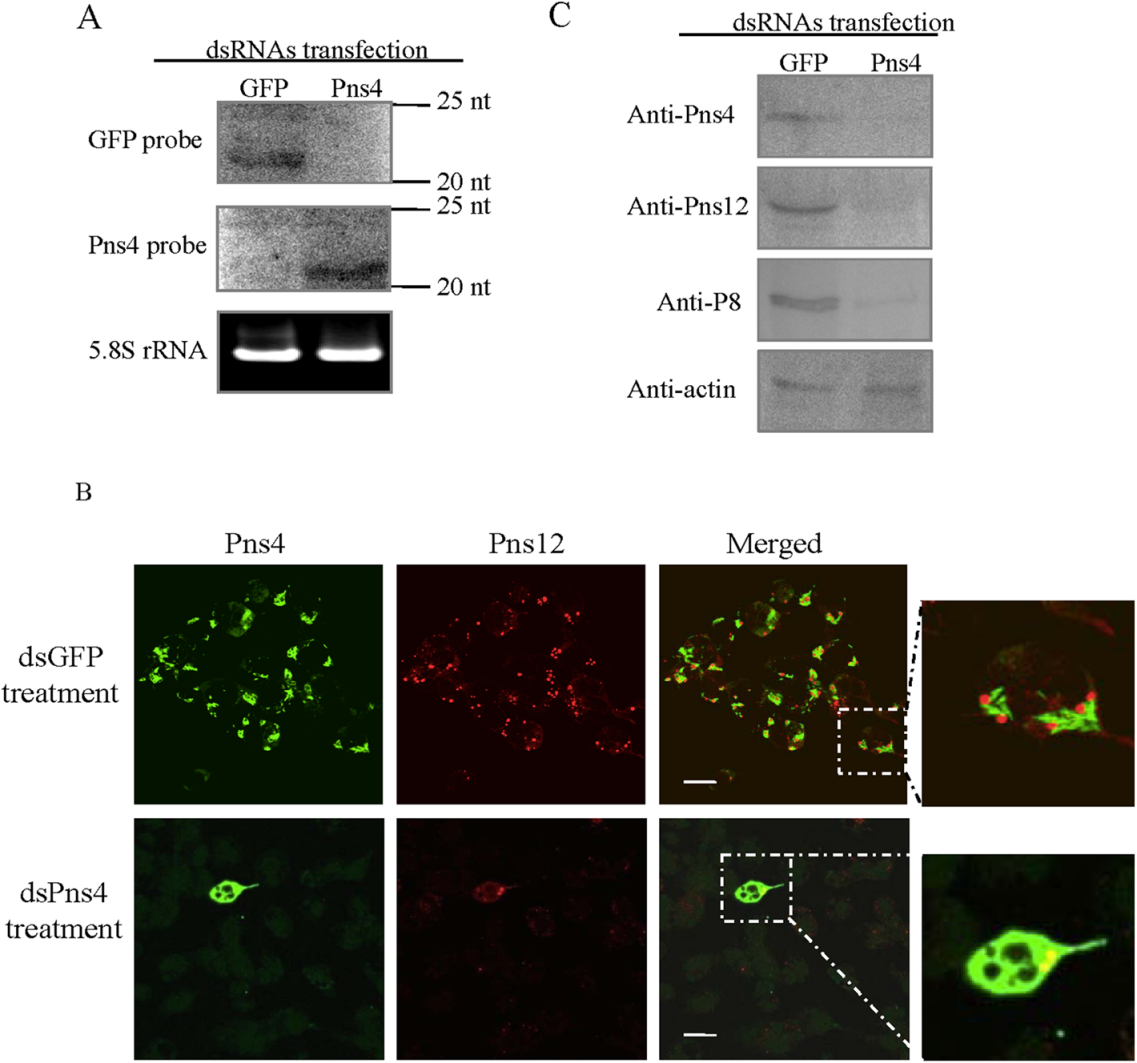
RNAi induced by dsPns4 inhibited RDV infection in cultured leafhopper cells *in vitro*. **a** siRNAs were active in the cultured leafhopper cells transfected with synthesized dsGFP or dsPns4 at 72 h post-transfection. 5.8S rRNA was used as a control to indicate loading of equal amounts of RNA in each lane. **b** The infection of RDV was blocked by RNAi induced by dsPns4 in cultured leafhopper cells. At 36 hpi, cells were immunolabeled with Pns4-FITC (green) and Pns12-rhodamine (red). The enlarged images displayed green fluorescence (Pns4-FITC) and red fluorescence (Pns12-rodanmine) of the merged images in the boxed areas in each panel, indicating that Pns4 distributed at the periphery of viroplasms. Bars, 20 μm. **c** The treatment of dsPns4 significantly reduced the synthesis viral proteins in cultured leafhopper cells with Western blotting assay at 36 hpi. Proteins separation by SDS-PAGE was performed to detect Pns4, Pns12 or P8 with Pns4-, Pns12- or P8-specific IgGs, respectively. Actin was used as a control and was detected with actin-specific IgG

In order to investigate the functional role of Pns4 of RDV, the RNAi activity induced by dsRNA specific for the Pns4 gene (dsPns4) was examined in cultured leafhopper cells. Cultured leafhopper cells were treated with dsPns4 and dsRNAs specific for the green fluorescence protein (dsGFP) by cellfectin-base transfection. It was confirmed that cellfectin treatment had no obvious influence on cellular viability and RDV infection in cultured leafhopper cells (data not shown). It has been previously suggested that RNAi is triggered by the appearance of small interfering RNAs (siRNAs) corresponding to the mRNA target sequence [20]. Northern blot assay demonstrated that siRNAs approximately 21 nt in length from dsPns4 and dsGFP treatments were detected (Fig. 1a), indicating that RNAi induced by dsRNAs was active in cultured leafhopper cells.

To investigate the effects of the knockdown of Pns4 expression on viral infection, cultured leafhopper cells growing on coverslips were transfected with dsRNAs, and inoculated by purified RDV. At 36 h post-inoculation (hpi), infected cells were fixed, immunolabeled with Pns4-specific IgG conjugated to fluorescein isothiocyanate (Pns4-FITC) and Pns12-specific IgG conjugated to rhodamine (Pns12-rhodamine), and observed with a confocal microscope, as described previously [26]. In cells transfected with dsGFP, viroplasm of Pns12 distributed in the cytoplasm (Fig. 1b). Abundant Pns4 appeared as minitubules which accumulated in the cytoplasm or at the periphery of the viroplam (Fig. 1b). In contrast, in cultured cells transfected with dsPns4, Pns4 was restricted in a limited number of infected cells, and the formation of viroplasm of Pns12 was almost completely blocked (Fig. 1b). These results suggested that the knockdown of Pns4 expression strongly impaired the formation of viroplasm, the machinery of viral replication, leading to the blocking of the subsequent viral infection.

For the purpose of further analyzing the effects of RNAi induced by dsPns4 on the formation of Pns4 minitubles and viroplasm, as well as viral assembly, western blotting assays were performed at 36 hpi with antibodies against viral nonstructural proteins Pns4 and Pns12, or the major outer capsid protein P8, to determine the expression level of corresponding proteins. Here, the expression level of Pns12 was served to judge for viroplasm accumulation, and the expression level of P8 was served to judge for RDV accumulation. As shown in Fig. 1c, the treatment of dsPns4 caused significant reduction of the accumulation of Pns4, Pns12 and P8. These results revealed that the knockdown of Pns4 expression inhibited viral replication possibly via affecting the development of the viroplasm. We deduced that Pns4 minitubules were essential for viral infection and possibly assisted the viropalsm to perform the function of replication and assembly of progeny virions.

### RNAi induced by dsPns4 inhibits the replication and infection of RDV in the insect vector

The efficiency of RNAi in cultured insect vector cells suggested that it was possible to introduce dsRNA into intact insects to investigate the role of Pns4 *in vivo*. The preliminary test showed that the microinjection with dsRNAs targeting RDV Pns4 gene caused no phenotypic abnormalities in leafhoppers (data not shown). The synthesized dsPns4 was microinjected into the second-instar nymphs of leafhoppers, which were then allowed a 3-day acquisition access period (AAP) on RDV-infected rice plants. At 12 days post-first access to diseased plants (padp), RT-PCR was conducted to detect the presence of the genes for Pns4 and P8 to analyze the viruliferous rate of insects. The result showed that about 50.7 % (*n* = 100, 3 repetitions) of leafhoppers microinjected with dsGFP were detected to contain Pns4 and P8 genes (Table 1). In contrast, about 15.6 % (*n* = 100, 3 repetitions) of leafhoppers microinjected with dsPns4 were detected to be positive for Pns4 and P8 genes.

**Table 1:** RNAi induced by dsPns4 significantly inhibited viral infection in insect vectors

Microinjection^a^	No. of insects giving positive results detected by RT-PCR (*n* = 100)		
	Expt I	Expt II	Expt III
dsGFP	51	50	51
dsPns4	16	15	16

^a^Second-instar nymphs of *N. cincticeps* were microinjected with dsGFP or dsPns4, allowed a 3-day AAP on virus-infected rice plants, and then placed on healthy rice seedling for 9 days

**Fig. 2:**
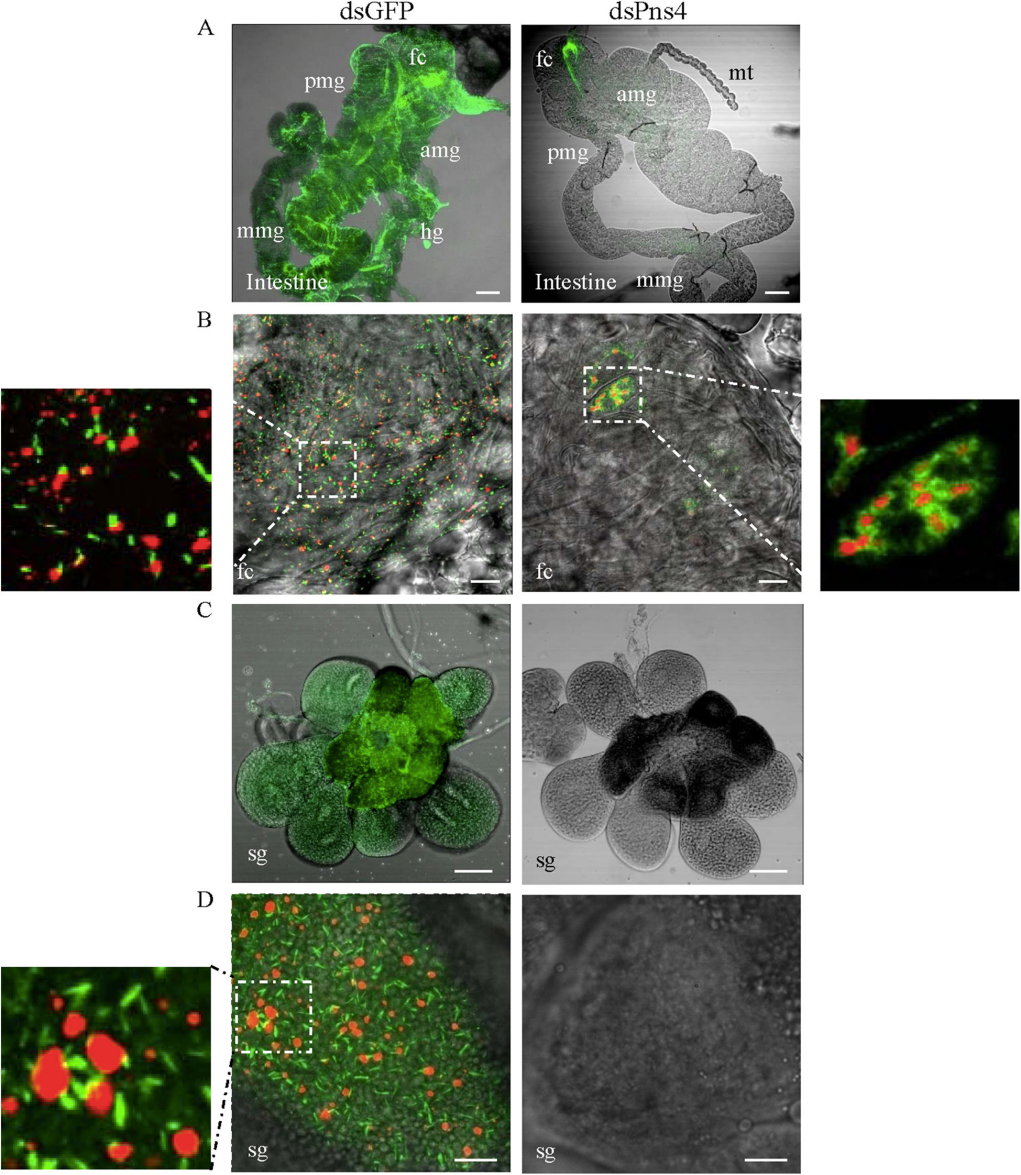
Microinjection of dsPns4 inhibited RDV infection and spread in insect vectors *in vivo*. At 12 days padp, the dissected intestines **a** and **b** and salivary glands (**c** and **d**) from leafhoppers receiving dsGFP or dsPns4 were immunolabeled with virus-FITC (green) (**a** and **c**), Pns4-FITC (green) (**b** and **d**) or Pns12-rodanmine (red) (**b** and **d**). The images with green fluorescence or red fluorescence were merged under a background of transmitted light. The enlarged images showing green fluorescence (Pns4-FITC) and red fluorescence (Pns12-rodanmine) of the merged images in the boxed areas in each panel, indicated that both of minitubules and diffusion of Pns4 distributed at the edge of viroplasms. fc, filter chamber; amg, anterior midgut; mmg, middle midgut; pmg, posterior midgut; hg, hindgut; mt, Malpighian tubules; sg, salivary gland. Bars, 100 μm (**a** and **c**) and 10 μm (**b** and **d**)

These results not only confirmed that Pns4 expression could be knocked down by dsPns4 microinjection, but also revealed that Pns4 contributed to efficient viral infection in leafhoppers. Then, the internal organs of leafhoppers (*n* = 30) microinjected with dsRNAs at 12 days padp were dissected, immunolabeled with Pns12-, Pns4- or virus-specific antibodies, and examined by immunofluorescence microscopy. The results showed that in leafhoppers microinjected with dsGFP, viral antigens distributed throughout the whole intestine and salivary gland in about 54 % of tested leafhoppers (Fig. 2a and c). Pns4 appeared as minitubules in the cells of intestine and salivary gland of viruliferous leafhopper (Fig. 2b and d). Some Pns4 minitubules distributed at the edge of viroplasms of Pns12 (Fig. 2b and d). In leafhoppers microinjected with dsPns4, RDV infection was restricted in a particular area of the filter chamber in the intestine in about 18 % of tested leafhoppers (Fig. 2a). Pns4 distributed diffusely in the cytoplasm and some were observed at the edge of the viroplasm within the epithelial cells of the filter chamber (Fig. 2b). No salivary gland was immunolabeled by Pns12-, Pns4- or virus-specific antibodies in the dsPns4-treated leafhoppers (Fig. 2c and d). These results indicated that the dsPns4 treatment specifically knocked down Pns4 expression in the initially infected sites of the filter chamber, so that RDV infection in the filter chamber was inhibited and viral spread to the salivary glands was blocked.

## Discussion

In the present study, we have advanced the previous study that Pns4 of RDV surrounded the viroplasms in a ring-like structure at the early stage of viral infection and it formed bundles of minitubules at later stages of viral infection in cultured insect vector cells [26]. It was found that when the expression of Pns4 was significantly reduced by RNAi induced by treatment of dsRNA from Pns4 gene, the development of viroplasms and viral replication were strongly inhibited both in cultured insect vector cells and the insect vectors (Figs. 1 and 2), revealing that Pns4 of RDV is required for viral efficient infection in insect vector. Because the non-structural protein Pns10 of RDV has been confirmed to assembly tubules to package virions for viral efficient spread in insect vectors, and virions are not directly associated with the minitubules of Pns4 in insect vectors [17–21, 26], it seemed that Pns4 was not involved in viral spread in insect vectors. The accumulation of the minitubules of Pns4 at the periphery of viroplasm, and the essential role of Pns4 in viral infection illustrated that Pns4 may contribute to viral replication or progeny virions assembly. Viroplasms are generally thought to be devoid of translational machinery ribosomes, so the synthesis of viral proteins was thought to occur in the cytosol [22]. We have observed that ribosomes were densely accumulated on the surface of minitubules of Pns4 at the peripheral of the viroplasms in insect vectors (Wei et al., unpublished data). Thus, it was assumed that the minitubules of Pns4 was involved in viral protein synthesis during virus replication and assembly in insect vectors.

In the family *Reoviridae*, non-essential genome segments particularly are prone to rearrangements and rearranged segments are stably maintained [27–30]. This is also true for phytoreoviruses such as would tumor virus and RDV [31–33]. Despite these facts, no rearrangement on S4s of the two phytoreoviruses have been reported, further confirming that Pns4 of phytoreoviruses was essential for viral infection.

As part of their infective strategy, viruses in *Reoviridae* family exploit various tubule structures to facilitate their replication and translocation. Viral nonstructural protein P7-1 of southern rice black-streaked dwarf virus (SRBSDV), a fijivirus, has intrinsic ability to assemble into tubules that package virions to spread along the actin cytoskeleton in insect vector cells [34]. The nonstructural protein NS1 of BTV, an orbivirus, is also able to constitute helically coiled ribbons to form tubules of 52.3 nm in diameter and up to 100 nm long [3, 35]. This shape is identical to Pns4 minitubules in morphology [26], though it is wider than Pns4 minitubules. Some studies reveal that these tubules are involved in the translocation of virions, cellular pathogenesis and morphogenesis of BTV, but the defined role of NS1 is yet to be determined [36]. In addition to viruses that are able to form tubule structures by their nonstructural proteins, there are also several viruses that exploit host microtubules to achieve their replication and trafficking of viral particles [37, 38]. For example, in rotaviruses, viroplasm assembly, fusion and structural maintenance are dependent on the microtubular network of the host [1]. Inside and adjacent to the viroplasm of reoviruses, coated microtubules are utilized to attach to mature virions and empty viral particles [4]. Further studies address that the μ2 protein of reoviruses, an essential viroplasm protein, binds microtubules and tethers viroplasms to the cytoskeleton of the host to determine the viral inclusion morphology [39, 40]. These data have inspired us to presume the minitubules of Pns4 may act a role as describe above.

Unlike viruses in the genus *Orbivirus*, *Reovirus* or *Rotavirus*, there is no reverse-genetics system in cultured cells for RDV, and methods to purify viroplasm, composite viroplasm, and assemble viral particle *in vitro* are absent. Therefore, a direct evidence of Pns4 playing a role in the assembly of viroplasm was unable to be obtained here. Further studies will be conducted to investigate the mechanisms underlying the structures of Pns4 in the progress of virus infection and the assembly of virions via the interaction of Pns4 with viral proteins and host components.

## Conclusions

The functional role of Pns4 in the viral infection cycle was investigated using the system of RNAi in cultured leafhopper cells *in vitro* and insect body *in vivo*. This study provides evidence that Pns4 is essential for viral infection and replication in insect vector. We also assume that Pns4 directly participates in executing the functional role of viroplasm for viral replication and assembly of progeny virions in insect vector.

## Methods

### Cells, viruses, vectors and antibodies

The nonviruliferous leafhopper vector *N. cincticeps* were collected in Fujian Province, China and propagated for several generations at 25 ± 3 °C in laboratory. Cultured cells, maintained in LBM growth medium, were originally developed from embryonic fragments of *N. cincticeps* [41]. The rice samples infected with RDV were initially collected from Yunnan Province, China, and propagated for several generations via transmission by *N. cincticeps*. Rabbit polyclonal antisera specific for Pns4, Pns12 and virus were prepared, as previously described [22]. IgGs were purified from specific polyclonal antisera, followed by conjugation directly to FITC or rhodamine according to the manufacturer’s instructions (Invitrogen). Finally, Pns4-/virus-FITC and Pns12-rhodamine were obtained for following immunofluorescence detection.

### dsRNAs synthesis *in vitro*

A T7 RNA polymerase promoter with the sequence 5’-ATTCTCTAGAAGCTTAATACGACTCACTATAGGG-3’ was added to the forward primer (5’ AGGCAGTACATCGTCACCC 3’) and reverse primer (5’ GCAATCACGCTCGCAAC 3’) at the 5’ terminal to amplify a region of about 815 bp of the Pns4 gene of RDV. The promoter was also added to 5’ terminal of the forward and reverse primers to amplify the full length of the GFP gene as a control. PCR products were transcribed into dsRNAs *in vitro* using the T7 RiboMAX (TM) Express RNAi System according to the manufacturer’s protocol (Promega). Purified dsRNAs were examined using agarose gel electrophoresis to insure their integrity and quantified by spectroscopy.

### Effects of synthesized dsRNAs on viral infection in cultured insect vector cells

Eight μg dsRNA and 6 μL cellfectin II Reagent (Invitrogen) were diluted individually in 50 μL LBM without fetal bovine serum and antibiotics, mixed gently together at room temperature for 40 min, and allowed to incubate with cultured cells for 8 h. Thereafter, cultured cells were inoculated with purified RDV at a high multiplicity of infection of 10 in a solution of 0.1 M histidine that contained 0.01 M MgCl_2_ (pH 6.2; His-Mg) at 25 °C for 2 h, and were finally recovered for complete culture [20].

At 36 hpi, cultured cells were fixed in 4 % paraformaldehyde in PBS for 30 min, and then penetrated in 0.2 % Triton-X for 10 min. Cells were immunolabeled using Pns4-FITC and Pns12-rhodamine for 45 min, then observed with an inverted confocal microscope (Leica TCS SP5).

### Effects of synthesized dsRNAs on viral infection in insect vectors

The nonviruliferous second-instar nymphs were microinjected with 0.1 μL of dsRNAs (0.5 μg/μL) at the intersegmental region of thorax, and then fed on RDV-infected rice plants for 3 days. The nymphs were then kept on healthy rice seedlings for 8 days. The intestines and salivary glands of the insects were dissected, fixed in 4 % paraformaldehyde in PBS for 2 h, penetrated with 2 % Triton-X for 1 h and incubated in Pns4-/virus-FITC, or Pns12-rhodamine for 2 h, as described previously [20]. The immunolabeled organs were observed with a confocal microscope to analyze the effect of dsRNAs on viral infection and spread.

### The detection of siRNAs by Northern blot analysis

Cultured cells were harvested for total RNA extraction using the TRIzol Reagent (Invitrogen) at 72 h post-treatment with dsRNA, in order to detect the presence of siRNAs specific for Pns4 or GFP genes in northern blots. Digoxigenin (DIG)-labeled ribo-probes corresponding to the negative-sense RNA of Pns4 or GFP genes were synthesized *in vitro* by T7 RNA polymerase using a DIG RNA Labeling kit (Roche). Approximately 5 mg of total RNA was probed using a DIG Northern starter kit (Roche) following the manufacturer’s protocol.

## Abbreviations

AAP: Days acquisition access period
BTV: Bluetongue virus
DIG: Digoxigenin
dsGFP: dsRNAs specific for the green fluorescence protein
dsPns4: dsRNA specific for the Pns4 gene
dsRNA: Double-stranded RNA
hpi: hours post-inoculation
padp: Post-first access to diseased plants
PBS: Phosphate buffered saline solution
Pns12-rhodamine: Pns12-specific IgG conjugated to rhodamine
Pns4-FITC: Pns4-specific IgG conjugated to fluorescein isothiocyanate
RDV: Rice dwarf virus
RNAi: RNA interference
RT-PCR: Reverse transcription-polymerase chain reaction
SDS-PAGE: Sodium dodecylsulfate polyacrylamide gel electrophoresis
siRNAs: Small interfering RNAs
SRBSDV: Southern rice black-streaked dwarf virus
virus-FITC: Virus-specific IgG conjugated to fluorescein isothiocyanate

## References

[1] Eichwald C, Arnoldi F, Laimbacher AS, Schraner EM, Fraefel C, Wild P (2012). Rotavirus viroplasm fusion and perinuclear localization are dynamic processes requiring stabilized microtubules. PLoS One.

[2] Guglielmi KM, McDonald SM, Patton JT (2010). Mechanism of intra-particle synthesis of the rotavirus double-stranded RNA genome. J Biol Chem.

[3] Boyce M, Celma CCP, Roy P (2012). Bluetongue virus non-structural protein 1 is a positive regulator of viral protein synthesis. Virol J.

[4] de Fernández Castro I, Zamora PF, Ooms L, Fernández JJ, Lai CM-H, Mainou BA (2014). Reovirus forms neo-organelles for progeny particle assembly within reorganized cell membranes. mBio.

[5] Attoui H, Mertens PPC, Becnel J, Belaganahalli S, Bergoin M, Brussaard CP, King AMQ, Adams MJ, Carstens EB, Lefkowitz EJ (2012). Family Reoviridae. Virus Taxonomy: Ninth Report of the International Committee for the Taxonomy of Viruses.

[6] Suzuki N, Sugawara M, Kusano T, Mori H, Matsuura Y (1994). Immunodetection of rice dwarf phytoreoviral proteins in both insect and plant hosts. Virology.

[7] Suzuki N, Sugawara M, Nuss DL, Matsuura Y (1996). Polycistronic (tri-or bicistronic) phytoreoviral segments translatable in both plant and insect cells. J Virol.

[8] Kano H, Koizumi M, Noda H, Mizuno H, Tsukihara T, lshikawa K (1990). Nucleotide sequence of rice dwarf virus (RDV) genome segment S3 coding for 114 K major core protein. Nucleic Acids Res.

[9] Suzuki N, Tanimura M, Watanabe Y, Kusano T, Kitagawa Y, Suda N (1992). Molecular analysis of rice dwarf phytoreovirus segment S1: interviral homology of the putative RNA-dependent RNA polymerase between plant-and animal-infecting reoviruses. Virology.

[10] Suzuki N, Kusano T, Matsuura Y, Omura T (1996). Novel NTP binding property of rice dwarf phytoreovirus minor core protein P5. Virology.

[11] Ueda S, Uyeda I. 1997. The rice dwarf phytoreovirus structural protein P7 possesses non-specific nucleotide acids binding activity *in vitro*. Molecular Plant Pathology. http://www.bspp.org.uk/mppol/1997/0123ueda/.

[12] Omura T, Ishikawa K, Hirano H, Ugaki M, Minobe Y, Tsuchizaki T (1989). The outer capsid protein of rice dwarf virus is encoded by genome segment S8. J Gen Virol.

[13] Yan J, Tomaru M, Takahashi A, Kimura I, Hibino H, Omura T (1996). P2 protein encoded by genome segment S2 of rice dwarf phytoreovirus is essential for virus infection. Virology.

[14] Zhong B, Kikuchi A, Moriyasu Y, Zhong B, Kikuchi A, Moriyasu Y (2003). A minor outer capsid protein, P9, of Rice dwarf virus[J]. Arch Virol.

[15] Zhou F, Wu G, Deng W, Pu Y, Wei C, Li Y (2007). Interaction of rice dwarf virus outer capsid P8 protein with rice glycolate oxidase mediates relocalization of P8. FEBS Lett.

[16] Zhou F, Pu Y, Wei T, Liu H, Deng W, Wei C (2007). The P2 capsid protein of the nonenveloped Rice dwarf phytoreovirus induces membrane fusion in insect host cells. Proc Natl Acad Sci U S A.

[17] Wei T, Kikuchi A, Moriyasu Y, Suzuki N, Shimizu T, Hagiwara K (2006). The spread of Rice dwarf virus among cells of its insect vector exploits virus-induced tubular structures. J Virol.

[18] Katayama S, Wei T, Omura T, Takagi J, Iwasaki K (2007). Three-dimensional architecture of virus-packed tubule. J Electron Microsc.

[19] Wei T, Shimizu T, Omura T (2008). Endomembranes and myosin mediate assembly into tubules of Pns10 of Rice dwarf virus and intercellular spreading of the virus in cultured insect vector cells. Virology.

[20] Chen Q, Chen H, Mao Q, Liu Q, Shimizu T, Uehara-Ichiki T (2012). Tubular structure induced by a plant virus facilitates viral spread in its vector insect. PLoS Pathog.

[21] Chen Q, Wang H, Ren T, Xie L, Wei T (2014). Interaction between nonstructural protein Pns10 of rice dwarf virus and cytoplasmic actin of leafhoppers is correlated with insect vector specificity. J Gen Virol.

[22] Wei T, Shimizu T, Hagiwara K, Kikuchi A, Moriyasu Y, Suzuki N (2006). Pns12 protein of Rice dwarf virus is essential for formation of viroplasms and nucleation of viral-assembly complexes. J Gen Virol.

[23] Chen Q, Chen H, Jia D, Mao Q, Xei L, Wei T (2015). Nonstructural protein Pns12 of rice dwarf virus is a principal regulator for viral replication and infection in its insect vector. Virus Res.

[24] Suzuki N, Watanabe Y, Kusano T, Kitafawa Y (1990). Sequence analysis of rice dwarf phytoreovirus genome segments S4, S5and S6: Comparison with the equivalent wound tumor virus segments. Virology.

[25] Uyeda I, Kudo H, Yamada N, Mastumura T, Shikata E (1990). Nucleotide sequence of rice dwarf virus genome segment 4. J Gen Virol.

[26] Wei T, Kikuchi A, Suzuki N, Shimizu T, Hagiwara K, Chen H (2006). Pns4 of Rice dwarf virus is a phosphoprotein, is localized around the viroplasm matrix, and forms minitubules. Arch Virol.

[27] Matthijnssens J, Rahman M, Van Ranst M (2006). Loop model: mechanism to explain partial gene duplications in segmented dsRNA viruses. Biochem Bioph Rse Co.

[28] Tanaka T, Sun L, Tsutani K, Suzuki N (2011). Rearrangements of mycoreovirus 1 S1, S2 and S3 induced by the multifunctional protein p29 encoded by the prototypic hypovirus Cryphonectria hypovirus 1 strain EP713. J Gen Virol.

[29] Tanaka T, Eusebio-Cope A, Sun L, Suzuki N (2012). Mycoreovirus genome alterations: similarities to and differences from rearrangements reported for other reoviruses. Front Microbiol.

[30] Troupin C, Dehée A, Schnuriger A, Vende P, Poncet D, Garbarg-Chenon A (2010). Rearranged genomic RNA segments offer a new approach to the reverse genetics of rotaviruses. J Virol.

[31] Nuss DL (1984). Molecular biology of wound tumor virus. Adv Virus Res.

[32] Pu Y, Kikuchi A, Moriyasu Y, Tomaru M, Jin Y, Suga H (2011). Rice dwarf viruses with dysfunctional genomes generated in plants are filtered out in vector insects: implications for the origin of the virus. J Virol.

[33] Murao K, Uyeda I, Ando Y, Kimura I, Cabauatan PQ, Koganezawa H (1996). Genomic rearrangement in genome segment 12 of rice dwarf phytoreovirus. Virology.

[34] Jia D, Mao Q, Chen H, Wang A, Liu Y, Wang H (2014). Virus-induced tubule: a vehicle for rapid spread of virions through basal lamina from midgut epithelium in the insect vector. J Virol.

[35] Hewat EA, Booth TF, Wade RH, Roy P (1992). 3-D reconstruction of bluetongue virus tubules using cryoelectron microscopy. J Struct Biol.

[36] Mathew A, Townsley E, Ennis FA (2004). Role of an arbovirus nonstructural protein in cellular pathogenesis and virus release. J Virol.

[37] Ohkawa T, Volkman LE, Welch MD (2010). Actin-based motility drives baculovirus transit to the nucleus and cell surface. J Cell Biol.

[38] Dodding MP, Way M (2011). Coupling viruses to dynein and kinesin-1. EMBO J.

[39] Parker JS, Broering TJ, Kim J, Higgins DE, Nibert ML (2002). Reovirus core protein mu2 determines the filamentous morphology of viral inclusion bodies by interacting with and stabilizing microtubules. J Virol.

[40] Ooms LS, Jerome WG, Dermody TS, Chappell JD (2012). Reovirus replication protein μ2 influences cell tropism by promoting particle assembly within viral inclusions. J Virol.

[41] Kimura I (1984). Establishment of new cell lines from leafhopper vector and inoculation of its cell monolayers with rice dwarf virus. P Jpn Acad.

